# Camera-Based Indoor Positioning System for the Creation of Digital Shadows of Plant Layouts

**DOI:** 10.3390/s23218845

**Published:** 2023-10-31

**Authors:** Julian Hermann, Konrad H. von Leipzig, Vera Hummel, Anton H. Basson

**Affiliations:** 1 Department of Industrial Engineering, Stellenbosch University, 145 Banghoek Rd., Stellenbosch 7600, South Africa; 2 ESB Business School, Reutlingen University, Alteburgstr. 150, 72762 Reutlingen, Germany; 3 Department of Mechanical and Mechatronic Engineering, Stellenbosch University, 145 Banghoek Rd., Stellenbosch 7600, South Africa

**Keywords:** digital shadow, plant layout, pan-tilt-zoom camera, AprilTags

## Abstract

In the past, plant layouts were regarded as highly static structures. With increasing internal and external factors causing turbulence in operations, it has become more necessary for companies to adapt to new conditions in order to maintain optimal performance. One possible way for such an adaptation is the adjustment of the plant layout by rearranging the individual facilities within the plant. Since the information about the plant layout is considered as master data and changes have a considerable impact on interconnected processes in production, it is essential that this data remains accurate and up-to-date. This paper presents a novel approach to create a digital shadow of the plant layout, which allows the actual state of the physical layout to be continuously represented in virtual space. To capture the spatial positions and orientations of the individual facilities, a pan-tilt-zoom camera in combination with fiducial markers is used. With the help of a prototypically implemented system, the real plant layout was captured and converted into different data formats for further use in exemplary external software systems. This enabled the automatic updating of the plant layout for simulation, analysis and routing tasks in a case study and showed the benefits of using the proposed system for layout capturing in terms of accuracy and effort reduction.

## 1. Introduction

Even though the concept of reconfigurable manufacturing systems was already introduced in the 1990s, the reconfigurability has become a growing necessity due to the increasing competitive pressure driven by unpredictable market demand, shorter product life cycles, greater product variety, lower production costs and higher environmental regulations. The development of modern production systems is also accelerated by the availability of new “Industry4.0” and internet of things technologies [1]. As a result of intensified competition, companies are increasingly concentrating on products with a high number of variants and small batch sizes. The production processes should be dynamically adapted in order to be able to react to various influences [2]. To achieve this, the concept of the so-called smart factories (SF) is pursued. As a requirement for SF, Mabkhot et al. [3] identified modular workstations in relation to flexible changes in the layout of the shop floor to adapt process functions. Xu and Hua [4] describe the dynamic reconfiguration of production lines as an element of SF, which requires a recording of the system landscape during operation.

The reconfiguration of the layout is typically not based on a predefined time interval. Instead, it depends on a variety of circumstances, including change in product mix, production volume fluctuations, technology upgrades, market dynamics and unforeseen system failures. A regular monitoring of the production performance as well as the circumstances allows to determine when a reconfiguration is meaningful in order to maintain efficiency and competitiveness [1,5].

As a starting point for a reconfiguration of production lines, it is necessary to determine the actual layout. According to Westkämper and Zahn [6], the planning process of a restructuring begins with an analysis and presentation of the existing factory. At this point the first difficulties already present themselves. The available plans of the existing factories and production facilities are in most cases outdated and modifications are seldom included in plans. The accuracy, speed and effort of conventional methods of actual recording do not correspond to the present and future requirements.

The aforementioned outdated plans not only cause difficulties in the layout planning tasks, they also affect material flow analysis and the route planning for material supply.

In order to track material flows in the factory, radio frequency identification (RFID) tags or barcodes are often used [7,8]. The tags or codes are attached to the objects to be tracked. These are scanned either on static readers or with portable devices at defined workstations. The position of the readers must therefore be known to ensure the location of the objects. If the readers’ positions changes and the plans are not updated, the collected data is of limited value. A similar situation applies to modern tracking systems used for material flow analysis. Real-time localisation systems (RTLS) are used to determine the approximate position of active tags attached to the objects to be tracked. Thus the paths of the objects can be traced through the production process and the positions that the objects waited at and for what duration can be identified. To assign the positions to workstations and machines, the layout of the production area must be known. Changes to the structure without corresponding updates of the plans render the subsequently generated data unusable, as the recorded positions cannot be clearly assigned to the infrastructure.

Another example of systems whose function is dependent on the plant layout are automated guided vehicles (AGV) and autonomous mobile robots (AMR), which are often used for the material transport [9]. The classic navigation methods of AGVs, such as mechanical, optical, or magnetic guidance tracks, are unsuitable in a dynamic environment. Thus, in dynamic environments, AMRs are often used. Unlike AGVs, which rely on a central unit for planning and routing, AMRs can communicate independently with other resources such as machines and systems, for example enterprise resource planning or control software, and can take autonomous decisions [10]. In addition, AMRs are able to understand their environment using built-in sensors and can therefore independently plan optimal routes between two points in a factory using intelligent algorithms. Therefore simultaneous localization and mapping (SLAM) techniques for mapping unknown areas are used [11]. In the SLAM process, the environment is scanned while the AMR is moving, which is usually done with 2D laser scanners. This creates a contour plan of the plant and the AMR orients itself to the geometrics of the surroundings. However, the SLAM method generally still finds its application in environments that do not vary over time or where changes can be modelled in advance [12]. However, as in real applications dynamic objects usually exist, which degrade the performance or lead to a failure of AMRs, it remains a challenge to use free navigating AMRs in dynamically changing operational spaces [13]. A digital shadow of the plant layout could thus contribute to improving AMR navigation.

## 2. Related Work

To keep the digital plant layout up-to-date, it is necessary to capture changes in the real layout and transfer it to the corresponding digital model. So far, there are few approaches for capturing the real layout. This section provides an overview of various methodologies adopted by researchers to address this challenge.

### 2.1. Previous Work by Other Researchers

A well-known process for capturing plant layouts is the use of 3D laser scanning technology. For this purpose, Westkämper and Zahn [6] propose a comprehensive five-step process using laser scanners. This process includes setting up a hall reference system, scanning the factory site, then analysing the acquired scan images, transferring the collected scan points into a computer-aided design (CAD) environment and finally creating realistic 3D models. Similarly, researchers at Chalmers University of Technology have developed a point-cloud-based strategy for creating virtual factory models [14]. This method uses a 3D laser scanner to capture a 360${}^{\circ }$ scan of the factory floor. Since there are obstacles such as machinery and equipment that could obstruct the laser beam, multiple scans are taken from different angles to ensure comprehensive coverage. These individual point clouds are then merged to create a holistic point cloud representation of the factory. This data fusion is followed by rendering processes that create immersive and photorealistic 3D environments that replicate the actual factory environment.

The Fraunhofer IFF developed a concept for the Matrix Fusion Factory. This describes a factory that is able to organise itself in the way it is needed. It uses modularised mobile machines that organise and position themselves [15]. To capture the current layout of the factory, 24 cameras were mounted on the ceiling, and 4 high-resolution cameras were mounted on a hall crane to take pictures while the crane is moving. The analysis of the images returns the positions of the machines via contour recognition [16].

A further project utilises digitally supported data recording. The geometrics are recorded manually by measurements, entered in an app on a tablet and then sent to a server. From there the data is provided by a central platform [17,18].

A quite different approach for a more specific use case for updating a digital layout is described by Braun et al. [19]. The focus is on the reconstruction of existing static production lines where industrial robots are involved. The layout of a production line is updated through an automated process using robot configurations. By extracting crucial robot information such as base positions, joining points, and process details, a software parser enables the reconstruction of stations. These positions are then transformed into a unified coordinate system, allowing for the connection of stations to form a complete production line.

Another concept for an automatically updated factory layout was presented by Lind et al. [20]. In this concept, sensors are attached to each facility to determine its position. In a test setup, the authors attached several sensors (H&D wireless sensors) with unspecified sensing technology to a rack system and determined the accuracy of the measured positions. As a result, a positional accuracy of worse than 2 m was determined, and the error in the orientation was not reported. Based on the evaluation, the authors pointed out that better and more accurate measurement systems are needed to create a digital twin of factory layouts.

### 2.2. Research Gaps, Shortcomings, and Limitations

In some digital twin concepts for entire factories, the recording of the plant layout is described as an important component [21,22,23,24]. So far, there are few approaches to capture a plant layout that use different sensing technologies. Although there is no precise definition of the required system parameters, some rough benchmarks for the performance of such a system have been given in the literature. For example, such a system should provide a unique identification and position data with an accuracy of a few centimetres for each facility. In addition, a capture process should be completed within a few minutes [25,26,27].

One of the most common methods is the use of a 3D laser scanner that is suitable for high-precision recording of the plant layout [28,29]. However, because it does not allow a fast recording and is difficult to handle, a laser scanner is often not ideal for measuring production environments [26,30]. In terms of the digital shadow of a plant layout, the laser scan method has some disadvantages due to the high manual effort required for each measurement, including preparation and post-processing and the resulting delay until the data is usable. A case study described that the scanning process takes less than 1 day for areas of between 400 and 1900 m${}^{2}$. The required time for post-processing was up to 20 h [31]. Scanning equipment and software licensing costs are also relatively high, at SEK 900,000 [32].

Using the manual measurement variant with app support is practical, but still requires a lot of manual effort for the measurement. In addition, possible errors in manual measurement cannot be excluded. Furthermore, as it is not certain whether the layout has been changed, measurements might be worthless.

The approach of the Matrix Fusion Factory offers a significantly higher sampling frequency. However, one camera can only cover a fixed area and thus a relatively large number of cameras must be used to cover the entire production area (and each one must be calibrated separately). The version with the indoor crane can certainly reduce the number of cameras considerably, but requires a crane that really covers the entire area. When evaluating the images using contour recognition, the case should also be taken into account that there are identical machine types that differ only in a small detail but cannot be clearly identified because the contours are too similar. For example, if several identical robots equipped with different end-effectors are used, these cannot take over each other’s tasks without conversion, but by using a pure contour recognition they would be classified in the same way.

In the example based on the extraction of robot positions, it can be seen that it is only applicable to static mounted systems, which have several robots integrated.

The approach of placing multiple sensors at each facility is more in line with the sense of a digital shadow, mainly because an automatic data flow between real and virtual components is being attempted. However, the authors have noted that the accuracy of the used system is questionable [33]. In addition, each facility must be equipped with active sensors, which increases costs and requires an separate energy source or extra charging management.

## 3. Proposed System

As a plant layout is defined by the poses (position and angular orientation) of the included facilities, it is necessary to use a sensor system that is able to detect these parameters. In order to create the captured plant layout in the sense of a digital shadow, it must be provided in a data format that can be used by external applications. Since a system for capturing the plant layout mainly deals with the position of the facilities, this is also referred to as an indoor positioning system (IPS). In the following, the architecture of such a system is explained and the sensory part of the proposed system is described in more detail.

### 3.1. Indoor Positioning System Architecture

**Figure 1:**
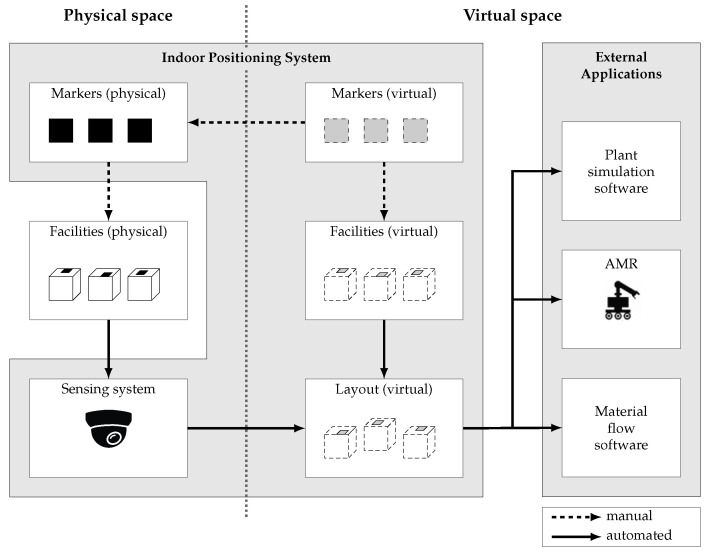
System architecture of the indoor positioning system.

The indoor positioning system and its interactions with surrounding systems and objects are illustrated in Figure 1. On the left side is the physical space, where the arrangement of the individual facilities constitutes the physical plant layout. On the right side is the virtual space, which includes a part of the IPS as well as external applications utilising the digital plant layout.

The idea of the proposed IPS is based on the use of a pan-tilt-zoom (PTZ) camera as a sensing system and fiducial markers as reference points, which are used to measure position and orientation and to identify individual facilities. The markers required in this approach are generated virtually in the IPS and added to the physical space by printing them out and attaching them to the physical facilities. The poses of the markers in the coordinate system of each facility are manually entered into the IPS. In this way, the markers are also linked to the digital models of the facilities in the virtual space. These models can be obtained from different sources in different sector-typical data formats and must be able to be imported. The previously described tasks must be carried out manually, but only once per facility.

Afterwards, the individual facility poses can be determined by the sensor system and automatically transferred to the virtual space. This enables the arrangement of the virtual plant models according to the real situation and thus leads to a digital representation of the plant layout. From this layout, the relevant data is extracted and converted into a suitable format so that the external applications can use it.

The group of external applications includes plant simulation software as well as routing software and material flow software. These toponyms describe systems which utilise the plant layout in different ways.

The functionality of the sensor subsystem as an important component of the overall system and the calibration as well as the performance measurement are described in the following subsections.

### 3.2. Sensor System Components

**Figure 2:**
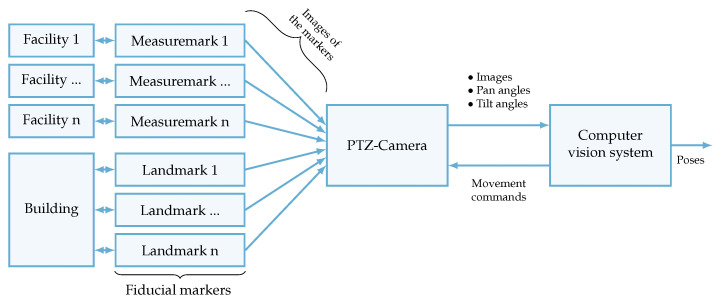
Overview of the sensory subsystem of the indoor positioning system.

Figure 2 shows an overview and the relation of the individual components that are used to capture the plant layout.

It is assumed that the building and the individual facilities are already available. The following components are subsequently added to capture the plant layout.

The fiducial markers are a special type of marker with a unique identification feature within a camera image that facilitates its accurate recognition and differentiation from other markers. In addition to its unique identification, it has the advantage of allowing measurement of its pose, which involves determining its exact position and orientation in relation to the camera. This capability enables spatial perception and measurement of the marker in the camera’s field of view. These markers are classified into the following two groups:

- **Measure marks:** The markers that are attached to the individual facilities to determine their pose and identity;
- **Landmarks:** The markers are attached to a fixed and known position in the plant coordinate system and serve as a reference.

The PTZ camera is a camera equipped with an image sensor that has a mechanism that allows it to be panned, tilted, and zoomed. The camera is preferably installed on the ceiling of a plant or in an elevated position to ensure a comprehensive view of all markers within its image. This configuration should provide optimal visibility and coverage of the markers from the camera’s perspective.

The computer vision system combines the capabilities of image analysis and camera control and enables simultaneous image processing and manipulation of the camera drives to achieve the desired results in the application.

### 3.3. Sensor System Overview

**Figure 3:**
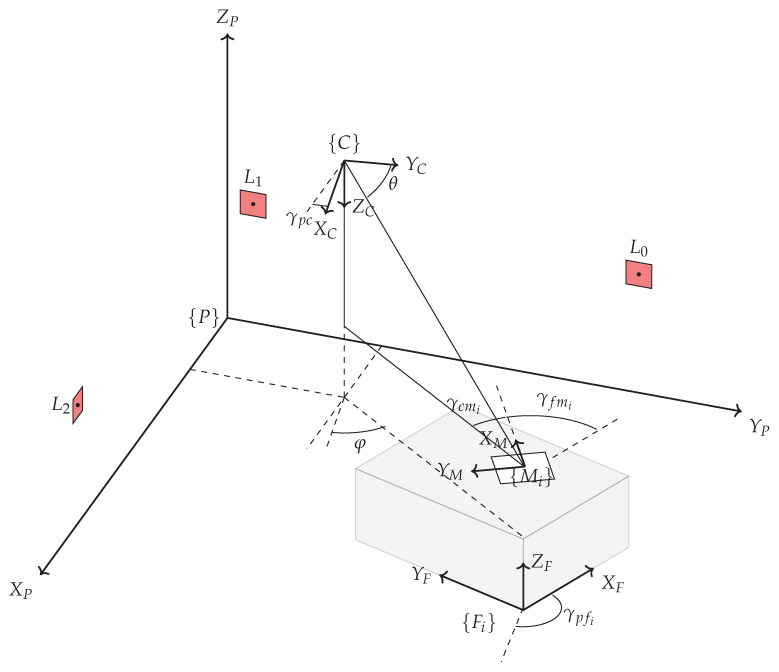
Arrangement of the sensor system components.

This section describes the arrangement of the system components and their mathematical relationships. The plant building contains the production system, i.e., all facilities such as machines, workstations, conveyor belts, etc., that are involved in the production process. The pose of each facility in relation to the plant coordinate system is the desired parameter to create the digital shadow of the plant layout. To describe the mathematical relationships using Figure 3, the following mathematical expressions are defined. The plant coordinate system {P} is used in the context of this study as a term for the world coordinate system. This represents a superordinate frame that serves as a reference coordinate system. The PTZ camera forms its own Cartesian coordinate system with the origin of the system at the centre of rotation of the pan and tilt axis and is represented by {C}. For the measure mark coordinate systems the designation {M} is used, and for the landmark coordinate systems the designation {L} is used. Since several markers of each group exist, they are numbered with the consecutive identifier *i*.

All facilities involved in the production process also have their own coordinate system {F}. As each of these is uniquely assigned to a measure mark, they also have an indicator *i* that matches the corresponding marker. The designations α,β,γ represent the rotation around the X,Y,Z axis of one coordinate system in relation to another. As designation for the pan and tilt angle of the PTZ camera, φ and θ are used.

First, the determination of the origin of the plant coordinate system is crucial. Although this origin should preferably be at a significant point in the building, such as a corner, it can be chosen at any suitable location. In Figure 3 the origin of the plant frame is marked by the symbol {P}. It is intended to represent the origin at a ground point of the building, with the Z axis as an indicator of height and the XY axes defining the floor of the building. The next element is the PTZ camera, which is mathematically represented by its own coordinate system {C}. The pose of this camera frame within the plant frame is defined by: ${}^{P}\xi_{C}=\left(x_{pc},y_{pc},z_{pc},\alpha_{pc},\beta_{pc},\gamma_{pc}\right)$, which contains the translation and rotation parameters used for the transformation between the two coordinate systems. The markers have a significant function in the measurement system. The pose of the marker, which includes both its position and orientation, is determined by image analysis of the camera image provided by the PTZ camera. To represent the marker’s coordinate system, a marker frame $\left\{M_{i}\right\}$ is used. As the marker is placed on a facility, it is located in the corresponding facility coordinate system, which is denoted by $\left\{F_{i}\right\}$. The relationship between these two coordinate systems is defined by the values of ${}^{F}\xi_{M}=\left(x_{fm},y_{fm},z_{fm},\alpha_{fm},\beta_{fm},\gamma_{fm}\right)$. Each facility frame within the plant frame possesses essential parameters that enable the reconstruction of the plant layout. These parameters consist of the position and orientation of each facility frame relative to the plant frame.

**Figure 4:**
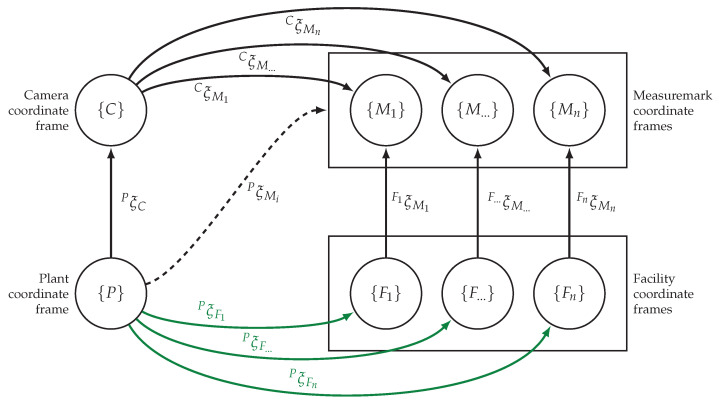
Pose-graph representation of the coordinate systems.

Figure 4 provides an overview of the interconnections between the various coordinate frames. The desired poses refer to the positions and orientations of facilities relative to the plant frame, therefore $P\xi_{F_{i}}$ is searched for. These desired poses are depicted as green lines in the figure. Since direct measurements of these desired poses are not feasible, an indirect approach is taken using the camera frame and individual marker frames to derive the facilities poses. By combining the relative poses of the camera frame and the marker frames, the desired poses can be determined. In this case, Equation (1) applies:

(1) $${}^{P}\xi_{F_{i}}={}^{P}\xi_{C}⊕{}^{C}\xi_{M_{i}}⊖{}^{F_{i}}\xi_{M_{i}}$$

### 3.4. Determination of the Camera Pose

The determination of the position and orientation of a scanner device (in this case the PTZ camera) based on the direction measurements is called resection within the field of geodesy. An overview of state-of-the-art methods on this topic is given by Awange and Paláncz [34] and Haralick et al. [35]. The described methods solve the fourth-degree polynomial equations for the calculation of the spatial resection based on Grunert [36] with different algorithms. All of these methods provide multiple solutions, and therefore, if one of these methods is used, a manual plausibility check is necessary to select the correct solution. They are unsuitable for this project because the calculation of the camera pose cannot be automated or can only be automated with difficulties and uncertainties.

**Figure 5:**
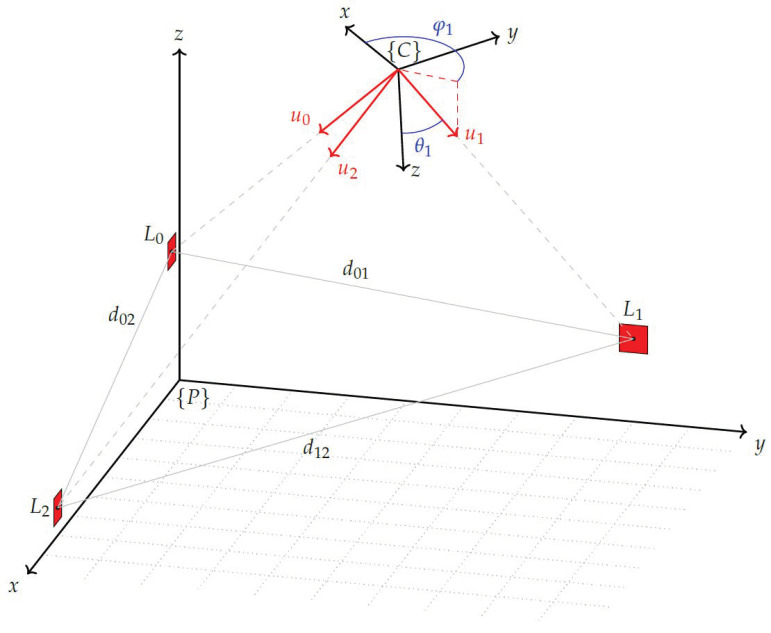
Parameters for pose determination of a PTZ camera.

A more recent approach to solving the resection problem is described by Donner [37], which is also based on directional measurements. It is pointed out that this method can also lead to ambiguous solutions, but only to wrong decisions if the Z-axis of the camera would be parallel to the XY-plane of the plant coordinate system. This situation does not occur if the camera is placed approximately parallel to the measurement plane as planned, and therefore, this method is more suitable for the measurement concept. Since the entire procedure will be tailored to specific complex problems in later steps, only the part up to the calculation of the positions of the landmarks in the camera coordinate system is used here. The prerequisite for the determination of the camera pose using this method is that the 3D coordinates of *n* landmarks (n≥3) in the plant coordinate system (${}^{P}\;L_{i}$) are known. In addition, the measured azimuth (pan) and elevation (tilt) angles ($\varphi_{i}$, $\theta_{i}$) of the camera to each of the landmarks are required. The following equations refer to the designations in Figure 5. This shows the plant coordinate system in which the camera coordinate system is located and three landmarks shown in red, which are located in both coordinate systems. The unit vectors $u_{0}$, $u_{1}$, and $u_{2}$ representing the viewing direction from the camera coordinate system to the individual landmarks are shown as red arrows.

In the first step, the Euclidean distances between the landmarks in space are calculated [37]. The known 3D positions of the landmarks are given by their XYZ coordinates, which are measured manually beforehand. Based on their spatial coordinates, the squared distances $d_{ij}^{2}$ between them can be calculated by applying Equation (2):

(2) $$d_{ij}^{2}={\left(x_{i}-x_{j}\right)}^{2}+{\left(y_{i}-y_{j}\right)}^{2}+{\left(z_{i}-z_{j}\right)}^{2}$$

fori∈Nandj∈Nandi≠j

The pan and tilt angles obtained from the camera are then used to calculate the direction vectors from the camera to the landmarks using Equation (3). The direction vectors are represented as unit vectors $u_{i}$.

(3) $$u_{i}=\left(\begin{array}{c} sin\left(\theta_{i}\right)cos\left(\varphi_{i}\right)\\ sin\left(\theta i\right)sin\left(\varphi_{i}\right)\\ cos(\theta_{i}) \end{array}\right)$$

By multiplying the vector $u_{i}$ with a scalar of unknown size $t_{i}$, the position of a landmark $L_{i}$ in the camera coordinate system can be obtained. The scaling factors, denoted as $t_{i}$, of the vectors $u_{i}$ are determined in such a way that the squared distances between their end points correspond to the squared distances $d_{ij}^{2}$ between the landmarks [37] according to Equation (2). This is accomplished by utilising the cosine theorem through the application of Equation (4) for each combination of landmark pairs $L_{i}$ and $L_{j}$. Here, the cosine of the spatial angle between the landmarks can be represented in a simplified way by $u_{i}\cdot u_{j}$, as they are unit vectors.

(4) $$d_{ij}^{2}=t_{i}^{2}+t_{j}^{2}-2t_{i}t_{j}u_{i}\cdot u_{j}$$

To obtain a solvable system of equations, at least three landmark pairs are required and therefore at least three landmarks must be used. Nevertheless, it is recommended to use more than three landmarks for spatial resection, which provide additional redundancy and improve the accuracy and robustness of the estimation [37]. In this case, an overdetermined system of equations can be obtained since more landmark combinations than number of unknowns emerge and the solution must be balanced using a least square method. To calculate the solutions for $t_{i}$, a numerical least-square solver was used in Python. Imaginary solutions and negative or zero multipliers were excluded and therefore unique solutions for $t_{i}$ are obtained. Multiplying the calculated $t_{i}$ values by the unit vectors $u_{i}$ results in the coordinates of the landmarks within the camera coordinate system according to Equation (5).

(5) $${}^{C}\;L_{i}=t_{i}u_{i}$$

After this step, the coordinates of the landmarks are known in both the plant and camera coordinate systems. From this point, Donner’s method is not continued and instead the Helmert transformation is used to determine the camera pose within the plant coordinates system. The Helmert transformation, also known as the seven-parameter transformation, is a coordinate transformation used in geodesy to convert three-dimensional Cartesian coordinates between different systems without distortion. It is therefore suitable for relating the camera coordinate system to the plant coordinate system. This transformation takes into account the translation, rotation, and scale differences between the two coordinate systems. For this measurement system, the corresponding Helmert transformation is shown in Equation (6).

(6) $${}^{P}\;L_{i}=\Delta +m\cdot R\cdot^{C}\;L_{i}$$

with

$$\begin{array}{ccccccc} {}^{P}L_{i}=\left[\begin{array}{c} {}^{x}p_{i}\\ {}^{y}p_{i}\\ {}^{z}p_{i} \end{array}\right], & \; & {}^{C}L_{i}=\left[\begin{array}{c} {}^{x}c_{i}\\ {}^{y}c_{i}\\ {}^{z}c_{i} \end{array}\right], & \; & \Delta =\left[\begin{array}{c} \Delta x\\ \Delta y\\ \Delta z \end{array}\right], & \; & R=R_{z}R_{y}R_{x} \end{array}$$

$$\begin{array}{ccccc} R_{x}=\left[\begin{array}{ccc} 1 & 0 & 0\\ 0 & cos\;\alpha & sin\;\alpha \\ 0 & -sin\;\alpha & cos\;\alpha \end{array}\right], & \; & R_{y}=\left[\begin{array}{ccc} cos\;\beta & 0 & -sin\;\beta \\ 0 & 1 & 0\\ sin\;\beta & 0 & cos\;\beta \end{array}\right], & \; & R_{z}=\left[\begin{array}{ccc} cos\;\gamma & sin\;\gamma & 0\\ -sin\;\gamma & cos\;\gamma & 0\\ 0 & 0 & 1 \end{array}\right] \end{array}$$

The transformation parameters, consisting of the rotation matrix R, the scaling factor *m*, and the translation vector Δ, are initially unknown. The translation vector Δ describes the displacement from the origin of the plant coordinate system to the origin of the camera coordinate system. For the rotation the matrices $R_{z}$, $R_{y}$ and $R_{x}$ are used, which rotate the camera coordinates system around the corresponding axis of the plant coordinates system. By inserting the now known positions of the landmarks within both coordinated systems (${}^{P}\;L_{i}$ and ${}^{C}\;L_{i}$) into Equation (6), an overdetermined system of equations is obtained. This is again resolved using a numerical least-square solver and the transformation parameters are determined. Thus, the pose of the camera inside the plant is clearly described by the position Δ and the rotation matrix R. The scale factor *m* would always be exactly 1 in case of an error-free measurement, but since variations are guaranteed to occur, it will deviate slightly.

### 3.5. Determination of the Measure Mark Positions

Before the measure marks can be transformed into the plant coordinate system using the calculated Helmert parameters, the position in the camera coordinate system must be determined based on the camera angles. The procedure that has been developed for this purpose is described below. The basic idea is to use the Helmert parameters and the landmarks to create a plane parallel to the bottom of the plant within the camera coordinate system. To obtain the coordinates of the measure marks, the intersection points between this plane and the direction vectors to each measure mark are determined in the camera coordinate system. Since it cannot be ensured that the landmarks are all placed at the same height from the ground of the plant, the first step is to create virtual landmarks that have the x and y coordinates of the real landmarks but the z coordinate is set to 0. These are inserted into Equation (7) and transformed into the camera coordinates using the Helmert parameters calculated in Section 3.4.

(7) $${}^{P}\;{\mathrm{VL}}_{i}=\Delta +m\cdot R\cdot^{C}\;{\mathrm{VL}}_{i}$$

**Figure 6:**
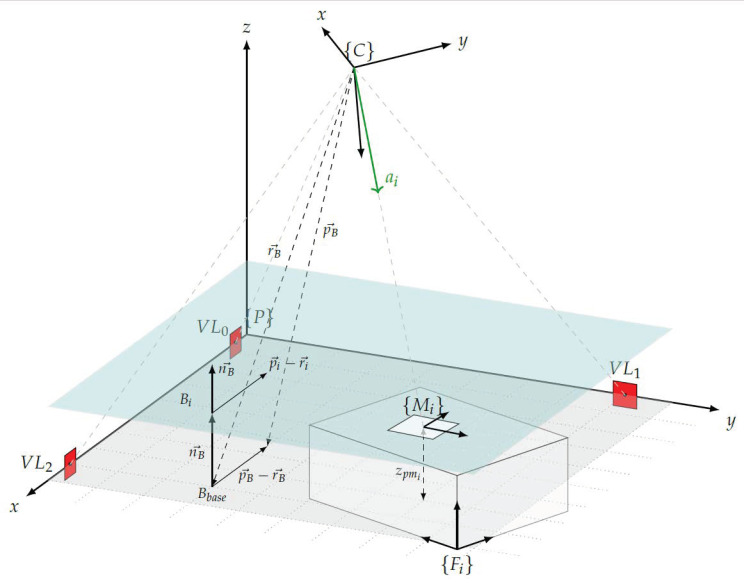
Parameters for position determination of a measure mark.

From the virtual landmarks in the camera coordinate system ${}^{C}\;{\mathrm{VL}}_{i}$, the surface *B* is constructed and presented in a point-normal form (see Equation (8)), indicated as a grey plane in Figure 6.

(8) $$B_{base}:\;\vec{n_{B}}\cdot \left(\vec{p_{B}}-\vec{r_{B}}\right)=0$$

where $\vec{n_{B}}$ represents the normal vector, which is a vector that is perpendicular to the plane. Vector $\vec{p_{B}}$ is a position vector pointing to any point on the plane, and $\vec{r_{B}}$ is a position vector pointing to a known point on the plane.

Now the direction to the measure marks is measured with the pan and tilt angles of the camera and displayed as direction vectors as in Equation (9).

(9) $$a_{i}=\left(\begin{array}{c} sin\left(\theta_{i}\right)cos\left(\varphi_{i}\right)\\ sin\left(\theta i\right)sin\left(\varphi_{i}\right)\\ cos(\theta_{i}) \end{array}\right)$$

In order to determine the position of the measure marks, it is first necessary to calculate a plane that is parallel to the plane $B_{base}$ but located at the height of the corresponding measure mark. To obtain this new plane, the plane $B_{base}$ is shifted along its normal vector $\vec{n_{B}}$ by the height of the marker on the facility $z_{pm_{i}}$. For each marker a new plane $B_{i}$ is created, which is calculated by Equation (10). The plane $B_{i}$ shifted parallel to the base is shown as a blue plane in Figure 6.

(10) $$B_{i}:\;\vec{n_{B}}\cdot \left(\vec{p_{i}}-\left(\vec{r_{B}}-\vec{n_{B}}\cdot z_{pm_{i}}\right)\right)=0$$

When moving the plane along its normal vector $\vec{n_{B}}$ by the translation vector $\vec{n_{B}}\cdot z_{pm_{i}}$, the normal vector $\vec{n_{B}}$ itself remains unchanged. The intersection point between the direction vector $a_{i}$ and the plane $B_{i}$ results in the coordinates of the corresponding measure mark ${}^{C}M_{i}$ in the camera coordinate system. This intersection point is calculated by using Equation (11).

(11) $${}^{C}M_{i}=\frac{\vec{n_{B}}\cdot \left(\vec{r_{B}}-\vec{n_{B}}\cdot z_{pm_{i}}\right)}{\vec{n_{B}}\cdot \vec{a_{i}}}\cdot \vec{a_{i}}$$

After the coordinates of the measure marks in the camera coordinate system ${}^{C}\;M_{i}$ have been calculated, the position in the plant coordinate system can be calculated by applying the Helmert transformation in Equation (12) with the previously determined transformation parameters.

(12) $${}^{P}\;M_{i}=\Delta +m\cdot R\cdot^{C}\;M_{i}$$

### 3.6. Determination of the Measure Mark Orientation

**Figure 7:**
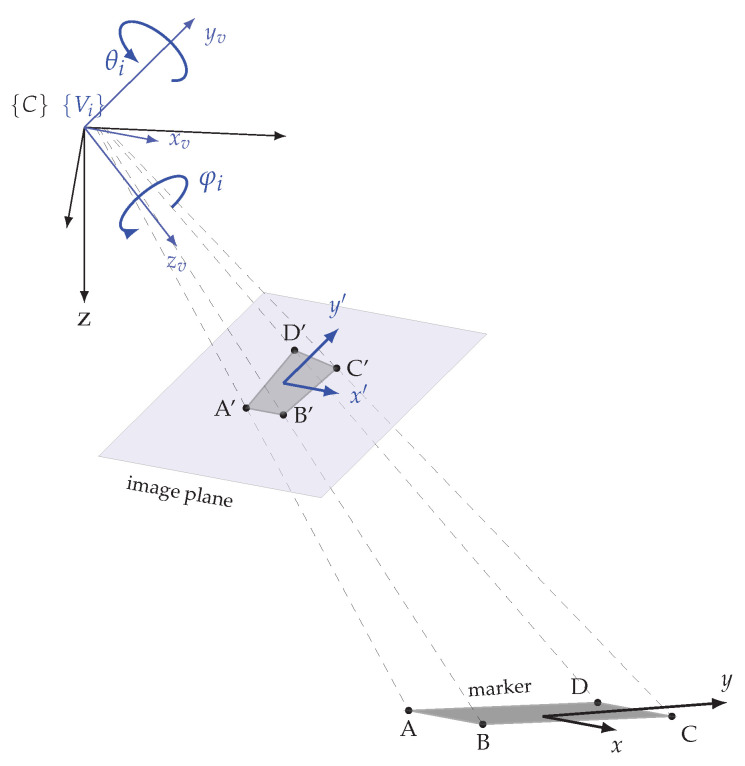
Projection of a marker onto the image plane.

Although the positions of the measure marks have already been calculated, the orientations of the markers in the plant coordinate system are still missing and are therefore considered in this section. The desired parameter is the rotation of the marker in relation to the plant coordinate system ($\gamma_{pm_{i}}$). The calculation of the rotation around the Z-axis of the marker is based on the use of the homography matrix. A homography matrix represents the geometric relationship between two planar surfaces. It defines a projective transformation between points in one plane and their corresponding points in another plane. In this case, the homography matrix describes the projective transformation between the coordinates of the marker in the physical world and the corresponding coordinates in the image plane [38,39]. This projection is shown in Figure 7.

The corner points A, B, C, and D are known in the marker coordinate system as the physical size was defined in advance. These corner points are mapped onto the image plane and their coordinates are detected in the image. The image plane is parallel to the XY plane of the viewpoint coordinate system $\left\{V_{i}\right\}$. That coordinate system has as its origin the optical centre of the camera, which is identical to the origin of the camera coordinate system {C}. As the camera sensor is moved by the pan and tilt function, the viewpoint coordinate system is rotated by the origin of the camera coordinate system for each pan angle $\varphi_{i}$ and tilt angle $\theta_{i}$ setting. The corner points $P_{j}$ on the marker plane (with *j* = A, B, C, D) as well as the corner points $P_{j}^{\prime }$ on the image plane can be described by Cartesian coordinates in the form $P_{j}={\left[x_{j},y_{j}\right]}^{T}$ and $P_{j}^{\prime }={\left[x_{j}^{\prime },y_{j}^{\prime }\right]}^{T}$. To simplify later calculations, the corner point coordinates are represented as homogeneous coordinates [40]. The homogeneous coordinates use an additional parameter *w* to represent the corresponding Cartesian point $\hat{P_{j}}={\left[\hat{x_{j}},\hat{y_{j}},\hat{w_{j}}\right]}^{T}$. To distinguish between homogeneous coordinates and Cartesian coordinates, the ^ symbol is used for homogeneous coordinates. The conversion of the homogeneous coordinates to Cartesian coordinates is done through division by *w*, as shown in Equation (13).

(13) $$\hat{P_{j}}=\left[\begin{array}{c} \hat{x_{j}}\\ \hat{y_{j}}\\ \hat{w_{j}} \end{array}\right]\;\Rightarrow \;P_{j}=\left[\begin{array}{c} \frac{\hat{x_{j}}}{\hat{w_{j}}}\\ \frac{\hat{y_{j}}}{\hat{w_{j}}} \end{array}\right]$$

The transformation between the virtual and physical points is described by Equation (14). Where *H* stands for the homography matrix, $\hat{P_{j}^{\prime }}$ are the points on the image plane and $\hat{P_{j}}$ are the points on the physical marker. To represent the Cartesian coordinates of the points in homogeneous coordinates, the parameter w=1 is applied [40].

(14) $$\hat{P_{j}^{\prime }}=H\hat{P_{j}}$$

(15) $$\left[\begin{array}{c} \hat{x_{j}^{\prime }}\\ \hat{y_{j}^{\prime }}\\ 1 \end{array}\right]=\left[\begin{array}{ccc} h_{00} & h_{01} & h_{02}\\ h_{10} & h_{11} & h_{12}\\ h_{20} & h_{21} & h_{22} \end{array}\right]\left[\begin{array}{c} \hat{x_{j}}\\ \hat{y_{j}}\\ 1 \end{array}\right]$$

The 3 × 3 homography matrix *H* has 9 unknowns, but as scalar multiples of homogeneous points are equivalent, *H* has only 8 degrees of freedom [41]. To enforce these 8 degrees of freedom, h22=1 is set, which fixes the scale of the matrix [38,42]. This allows to solve the resulting equation system to *H* using the four corner point pairs with the direct linear transformation (DLT) algorithm [43]. Under the assumption that all points on the marker are on a planar surface, i.e., z=0, a 3 × 3 homography matrix can also be written as product of the intrinsic camera parameter matrix *K*, the extrinsic camera parameter matrix [R|T] and the scaling factor *s*, as shown in Equations (16) and (17) [41].

(16) H=sK[R|T]

(17) $$\left[\begin{array}{ccc} h_{00} & h_{01} & h_{02}\\ h_{10} & h_{11} & h_{12}\\ h_{20} & h_{21} & h_{22} \end{array}\right]=s\left[\begin{array}{ccc} f_{x} & 0 & c_{x}\\ 0 & f_{y} & c_{y}\\ 0 & 0 & 1 \end{array}\right]\left[\begin{array}{ccc} R_{00} & R_{01} & T_{x}\\ R_{10} & R_{11} & T_{y}\\ R_{20} & R_{21} & T_{z} \end{array}\right]$$

The known parameters are the homography matrix *H* as well as the focal lengths ($f_{x}$,$f_{y}$) and the principal point ($c_{x}$,$c_{y}$). In order to calculate the rotation matrix *R* and the translation vector *T*, the scaling factor *s* must be defined beforehand. The magnitude of *s* can be restricted as the columns of the rotation matrix must all have the same magnitude. Since the rotation matrix consists of two columns, *s* is calculated as the average of the two columns magnitudes [39]. Since the DLT and the normalisation do not necessarily lead to a strictly orthonormal rotation matrix, for correction the polar decomposition of the rotation matrix is calculated [39,44]. For the intended application, only the rotation of the marker around its own Z-axis in the camera coordinate system {C} is required, this rotation value is denoted by $\gamma_{cm_{i}}$. To calculate this, first the marker rotation around the Z-axis of the viewpoint coordinates system $\left\{V_{i}\right\}$ is calculated from the rotation matrix with the arctan2 function. To transfer this rotation angle into the camera coordinate system {C}, the pan angle $\varphi_{i}$ has to be added. The reason is that the rotation of the image plane around the z-axis of the camera coordinate system is only influenced by the pan angle. Equation (18) shows the calculation of the rotation angle $\gamma_{cm_{i}}$.

(18) $$\gamma_{cm_{i}}=\varphi_{i}+arctan2\left(R_{i_{10}},R_{i_{00}}\right)$$

Subsequently, the rotation of the marker in the plant coordinate system $\gamma_{pm_{i}}$ can be calculated by adding the Z-rotation of the camera coordinate system around the plant coordinate system $\gamma_{pc}$, according to Equation (19).

(19) $$\gamma_{pm_{i}}=\gamma_{pc}+\gamma_{cm_{i}}$$

Through the previously performed calculation steps, the pose of the marker within the plant coordinate system is determined using the camera coordinate system and is described by Equation (20).

(20) $${}^{P}\xi_{M_{i}}={}^{P}\xi_{C}⊕{}^{C}\xi_{M_{i}}=\left(x_{pm_{i}},y_{pm_{i}},z_{pm_{i}},\gamma_{pm_{i}}\right)$$

### 3.7. Determination of the Facilities Poses

In the last step, the pose of a marker on the corresponding facility is taken into account to calculate the pose of the facility in respect to the plant coordinates system. The pose of the marker within the facility coordinate system is measured when the marker is attached and described by the XYZ coordinates and the rotation around the Z axis in the form ${}^{F_{i}}\xi_{M_{i}}=\left(x_{fm_{i}},y_{fm_{i}},z_{fm_{i}},\gamma_{fm_{i}}\right)$.

(21) $${}^{P}\xi_{F_{i}}={}^{P}\xi_{C}⊕{}^{C}\xi_{M_{i}}⊖{}^{F_{i}}\xi_{M_{i}}={}^{P}\xi_{M_{i}}⊕{}^{M_{i}}\xi_{F_{i}}$$

(22) $${}^{P}\xi_{F_{i}}=\left[\begin{array}{c} x_{pf_{i}}\\ y_{pf_{i}}\\ z_{pf_{i}}\\ \gamma_{pf_{i}} \end{array}\right]=\left[\begin{array}{c} x_{pm_{i}}-\left(x_{fm_{i}}\cdot cos\left(\gamma_{pf_{i}}\right)-y_{fm_{i}}\cdot sin\left(\gamma_{pf_{i}}\right)\right)\\ y_{pm_{i}}-\left(x_{fm_{i}}\cdot sin\left(\gamma_{pf_{i}}\right)-y_{fm_{i}}\cdot cos\left(\gamma_{pf_{i}}\right)\right)\\ z_{pm_{i}}-z_{fm_{i}}\\ \gamma_{pm_{i}}-\gamma_{fm_{i}} \end{array}\right]$$

By applying the pose algebra in (21), one obtains the Equation (22) for calculating the facility pose in relation to the plant coordinate system.

## 4. Materials and Methods

In order to conduct experimental tests with the previously described system concept, the following materials were used. For the sensor system, a PTZ camera type M5525-E from the company Axis (Lund, Sweden) was used. The camera’s features include a 360° endless pan angle and a 90° tilt angle. Both axes can be moved with a speed of 1.8° to 150° per second. The camera has a 10× optical and a 12× digital zoom and a maximum resolution of 1920 × 1080 pixels. In addition, the camera manufacturer provides an open and well-documented interface to the camera using the VAPIX^®^ and ONVIF^®^ protocols.

As fiducial markers, AprilTags are used, which enable a very stable recognition rate as well as an already implemented algorithm for detection and identification [38,45]. For the experiments, AprilTags of type 52h13 in a size of 100 mm are used.

Before the facility poses are measured, the accuracy of the camera’s axis is determined and the camera is calibrated.

### 4.1. Calibration

**Figure 8:**
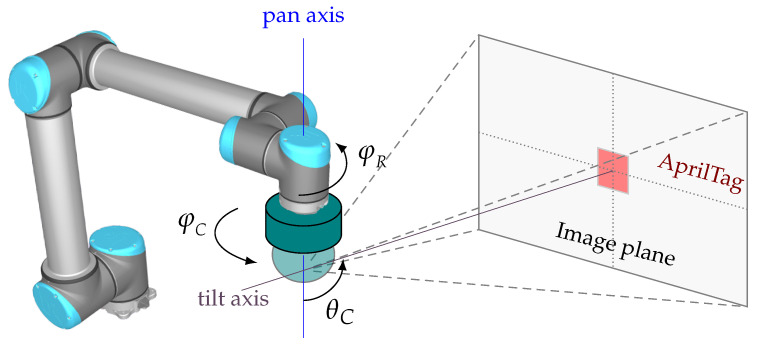
Test setup for the calibration of the camera axes.

First, the camera was attached to an industrial robot in a test setup, as schematically shown in Figure 8. An AprilTag was placed at a distance of 2 m from the camera so that it was in the camera’s field of view. This is used as an orientation point for the measurement and the robot axis as a reference for the angle of rotation.

First, the setup was used to determine the accuracy of the pan axis by moving the pan axis of the camera to the angle setting 0° in the positive direction and then adjusting the robot axis so that the centre of the AprilTag was in the centre of the camera image.

**Figure 9:**
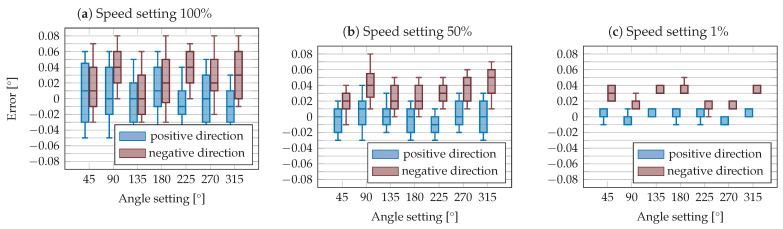
Error in cameras pan angle in positive and negative rotation direction with speed settings of (**a**) 100%, (**b**) 50%, and (**c**) 1%.

In the measurement, the robot axis $\varphi_{R}$ was moved in the negative direction and then the pan axis of the camera was moved in the positive direction until the image centre and the AprilTag centre coincided again. The chosen angle settings were 45° to 315° in steps of 45° and the speed settings 100%, 50%, and 1%. The procedure was as follows, explained using a numerical example. The robot axis was rotated by 45° in the negative direction. Afterwards, the pan axis was set in positive direction to the target value of 44.9° and subsequently incremented in steps of 0.01° until the image and AprilTag centres intersected. As a result, the difference between the set pan angle and the reference angle of the robot axis was saved. The procedure was repeated 50 times for each angle position and speed setting, and the same procedure was carried out in the reverse direction. In Figure 9, the results of the measurement series are shown with separate diagrams for each speed setting used.

Comparing the results between positive and negative direction of rotation, relatively large differences can be seen. Across all measurements, there is a difference of up to 0.04° between the median values, indicating that the backlash corresponds to this size. It is also obvious that the precision at a low speed setting is significantly better than at a higher speed setting. At a speed setting of 100% the error range is 0.11°, while at a speed setting of 1% the error range is just 0.02°. As a result of this experiment, two important points can be summarized. One is that the rotation direction of the pan axis resulting from the backlash is a source of relatively high inaccuracies. Secondly, the accuracy depends very strongly on the selected speed setting. This means that the error can be minimized if the camera always moves to the AprilTags in the positive direction and the speed is reduced to 1% in incremental search mode. Thus, the accuracy of the pan angle can reach ±0.01°.

In principle, one sees similar aspects with the tilt axis as with the pan axis. A reference run is also performed at the power connection, and this can also be triggered at any time via the API. In addition, the axis is driven by a stepper motor via a toothed belt and the angular speeds are also the same. The tilt of the camera can be adjusted over a range of 90° with a resolution of 0.01°. At a setting of 0° the tilt axis is orthogonal to the pan axis and at a setting of −90° parallel to the pan axis.

The same setup as before was used to calibrate the tilt axis. However, the camera was tilted by the robot by setting the intersection point of the camera axes as the tool centre point of the robot and rotating it around this point accordingly. Then, analogous to the previous experiment, different angular positions were approached by the robot and adjusted by moving the camera in the opposite direction. As for the speed setting in the incremental search mode, only 1% was chosen. This is because the camera only accepts one command for the speed of both axes. Since it has already been established that 1% must be set for high accuracy of the pan axis, this automatically applies to the tilt axis as well.

To begin with, the tilt setting was set to −90° and then the ptz camera’s pan axis was rotated to check if the axes are parallel. It could be seen that the image did not rotate around its centre. This eccentric rotation indicates an offset, the value of which was determined by moving the axis in 0.01° steps until the camera rotated around the centre of the image. The resulting offset of 0.06° was taken into account in the following measurements. For the calibration, the angle settings −90° to 0° in 5° steps were used. These were again approached from positive and negative directions and the difference from the reference value was recorded as an error. Each angle and direction setting was repeated 50 times in series. The tilt angle setting of −90° was used as the starting position for the measurement. From there, a slightly smaller angle was set for measuring each angle setting in the positive direction and then increased in 0.01° increments until the centre of the AprilTag was again in the centre of the image. For the measurement in negative direction, a slightly higher angle was set and then decreased in 0.01° steps until the target position was reached.

**Figure 10:**
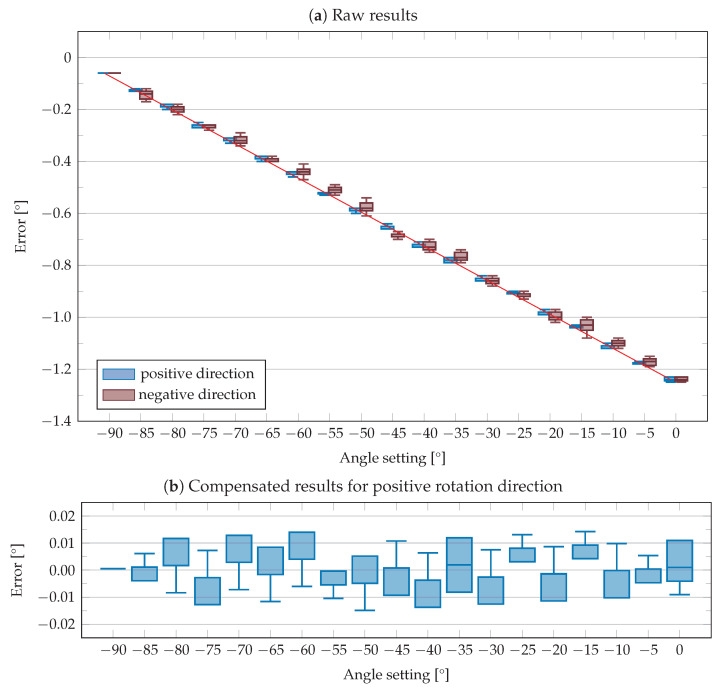
Error in cameras tilt angle (**a**) as raw measurement and (**b**) after compensation.

The raw measurement results are shown in Figure 10a. It can be seen that the tilt angle is subject to a systematic error, in the negative as well as in the positive moving direction. In addition, Figure 10a shows that the error range is higher in the positive rotation direction than the negative rotation direction. Therefore, the positive direction of rotation should be used in order to minimise the error. To correct the systematic error and to determine the real tilt angle from the set tilt angle, a regression analysis was applied using the raw measured values. This results in Equation (23) for the compensation of the systematic error.

(23) $$\theta_{real}=\theta_{set}+0.0132\cdot \theta_{set}+1.241$$

The regression line is also shown as a red line in Figure 10a. After applying the compensation to the raw measured values, the compensated results are obtained, which are shown in Figure 10b.

### 4.2. Test Setup

**Figure 11:**
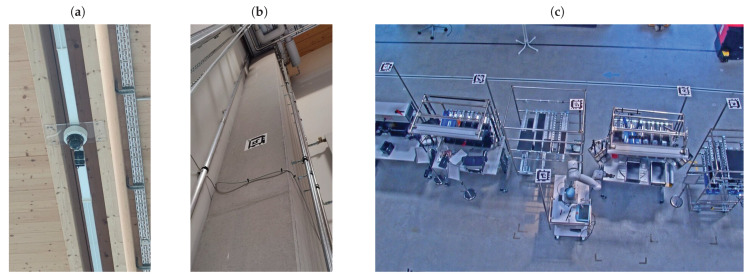
Preparation of the test setup with (**a**) the PTZ camera mounted at the ceiling, (**b**) a landmark attached to a column, and (**c**) facilities with attached measure marks.

After the camera axes have been calibrated, the test setup for determining facility poses within a factory building follows. Initially, the camera was installed at the ceiling of the building. It is mounted at a height of approximately 7 m above the floor, as shown in Figure 11a. Subsequently, 5 landmarks were mounted on the walls and columns of the building in positions at a height of approximately 3 m, where they were fully visible for the camera (see Figure 11c). These relatively simple steps are followed by the greater effort of attaching the markers to the individual facilities. Figure 11c shows some facilities with measure marks at the top as seen from the PTZ camera.

**Figure 12:**
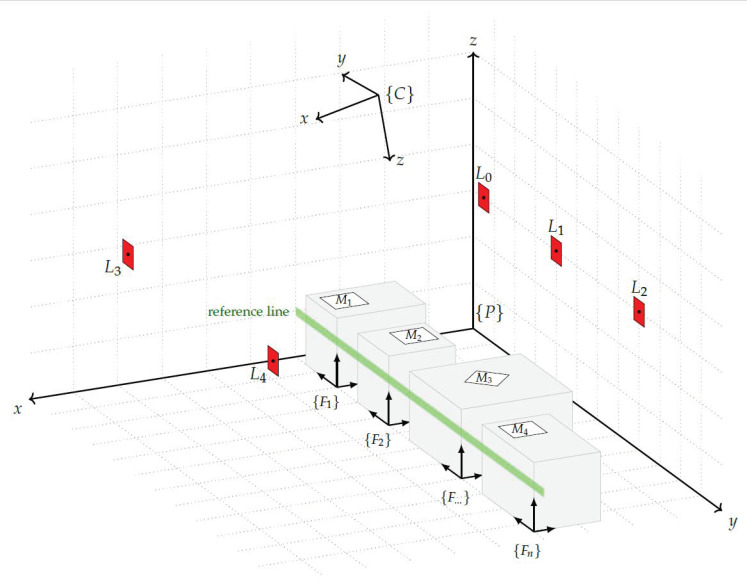
Test setup for the facility pose accuracy measurement.

After the preparations have been completed, the actual experimental setup illustrated in Figure 12 follows. For this purpose, a reference line was placed parallel to the Y-axis of the plant coordinate system by passing a string through the building. Along this reference line, 8 different facilities were aligned with a distance in the Y-direction of 2.5 m between the origin of their coordinate systems. The orientation results from the arrangement of the facilities on the reference line should be 180° for all facilities in relation to the plant coordinate system.

### 4.3. Measurement Procedure

**Figure 13:**
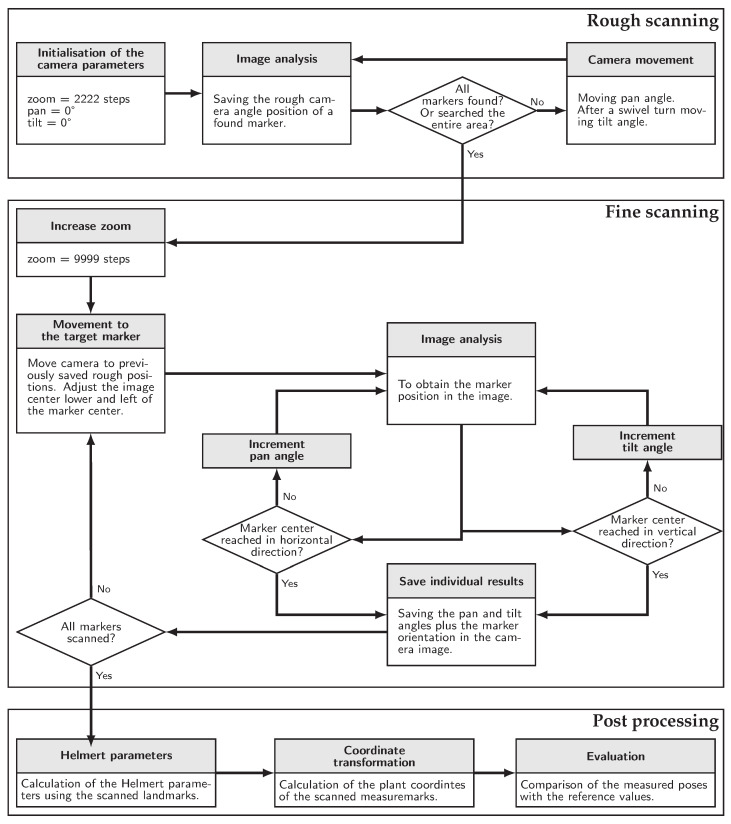
Measurement process flow.

The measurement procedure is explained using Figure 13, starting with the so-called rough scanning process. First, a low zoom level of 2222 steps is set on the camera and each axis is rotated to 0°. To do this, the corresponding parameters are sent from the computer vision system to the camera via the REST interface. The most recent image delivered by the camera via the MJPEG stream is then read and analysed using the AprilTag algorithm. If one or more markers are detected in the image, the image is centred on the marker one after the other and the respective pan and tilt positions of the axes are read out and saved.

If at this point not all markers have been found or the entire area has not been searched, the camera will be rotated further around the pan axis by the image aperture angle in the current zoom setting. When the pan axis has swivelled a full rotation, the tilt axis will turn further vertically. After all markers have been found in the rough scan or the entire area has been searched, the fine scan follows, where a higher zoom level is initially set. Subsequently, all positions stored in the rough scan are moved to in a loop. First a position is set to the lower left of the marker centre so that the gear backlash does not cause an error and then the pan and tilt settings are incremented until the image centre is in the marker centre. These camera angle settings, as well as the orientation of the marker in the image, are saved as a fine scanning result for the corresponding marker ID and then the next marker is scanned. After the fine scanning process has been completed for all markers, the data evaluation follows in post processing. To calculate the Helmert parameters, the markers defined as landmarks are used. This requires the coordinates measured in advance in the plant coordinate system and in the camera coordinate system, which are calculated from the measured pan and tilt angles using Equations (2)–(5). Then the coordinate transformation is performed with the measure marks, and thus the coordinates in the plant coordinate system are determined. Based on this, the pose of each facility in the plant coordinate system is calculated using Equation (22).

To evaluate the data, the Euclidean distance in the XY plane between the determined plant coordinates and the reference values is calculated, which provides the position error of the measuring system. In addition, the error in the rotation measurement of the facilities around the plant coordinate system’s Z-axis is determined.

### 4.4. Measurement Results

**Figure 14:**
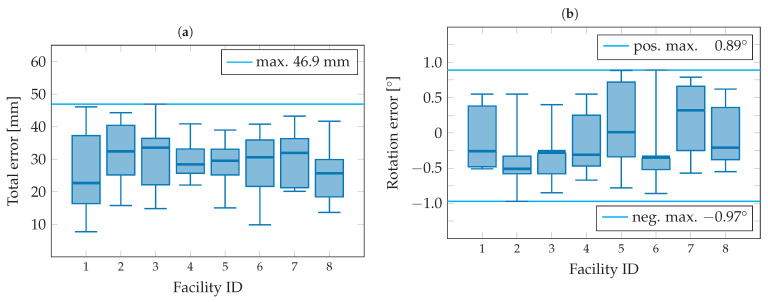
Error in the measurement of the facility poses shown with (**a**) the position error in XY plane and (**b**) the rotation error.

The result of the measurement series for determining the facility poses is shown in Figure 14. A maximum position error of 47 mm was achieved in the XY plane, as can be seen in Figure 14a. The maximum error of the facility rotation with respect to the plant coordinate system is in the range of approximately −1° to 0.9°, as shown in Figure 14b. This relatively high accuracy in the facility pose measurement should be sufficient to create a digital representation of the plant layout in adequate quality.

The time required for the scanning process was also determined. A distinction was made between the relatively constant rough scanning process and the fine scanning process, which depends on the number of markers in the system.

**Figure 15:**
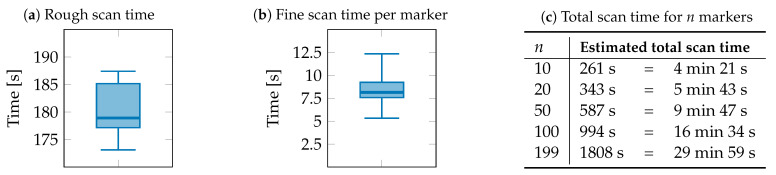
Time consumption for the scanning processes with (**a**) rough scan time, (**b**) fine scan time per marker, and (**c**) estimated total scan time.

The post-processing of the data, i.e., the calculation of the Helmert parameters and the coordinate transformation, was not taken into account as these times are negligibly small. Figure 15a illustrates the time required for the rough scan, which is about 180 s on average. However, due to occasional delays caused by drive commands over the Ethernet network and variations in image analysis times, there are slight variations of a few seconds. The time measurements for the fine scan per marker are shown in Figure 15b. They range from 5.32 s to 12.35 s with an average of 8.14 s to detect the position of one marker in the fine scanning process. Using these average values and extrapolating the total scan time for a given number of markers in the system, the table in Figure 15c is obtained. With 200 markers, the scanning process takes approximately 30 min, and with more markers, it correspondingly takes longer.

## 5. Case Study Validation and Results

This section describes the intended application of the digital shadow of a plant layout created with the proposed IPS. To determine the added value of the IPS, a production scenario was set up and the process of recording the layout was considered from the perspectives of factory planners, material flow analysts, and route planners. These stakeholders are dependent on a correct representation of the plant layout but they typically work with different software tools. Therefore, the IPS was designed to provide the captured layout in different data formats for exemplary software applications, allowing all stakeholders to work on the same data basis.

### 5.1. Digital Shadow of a Plant Layout from Different Perspectives

**Figure 16:**
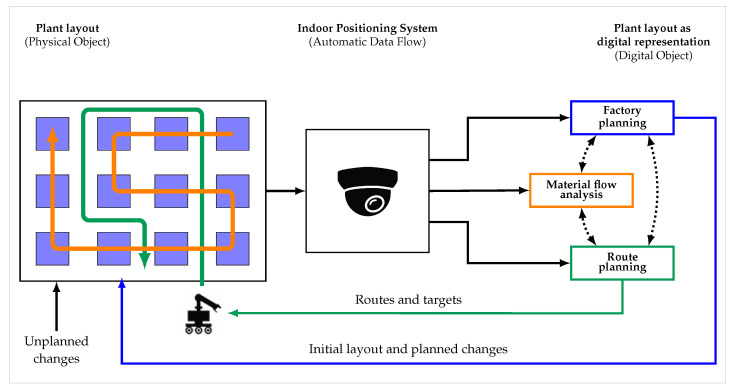
Digital shadow of a plant layout with different stakeholder perspectives.

The different stakeholder perspectives are illustrated in Figure 16, which shows the context of a plant layout in terms of the digital shadow.

On the left side a plant layout is shown, which results from the arrangement of different facilities, represented by the blue boxes. This production system is initially planned but is subject to planned and unplanned changes. With dedicated processes at these facilities, a product moves through the plant during its production process. This material flow is indicated by the orange line. In addition, there are transport systems such as AMRs that need to be adjusted to a given layout in order to find a way through the resulting labyrinth and perform delivery orders along their route, as represented by the green line.

### 5.2. Use Case Scenario

To validate the concept of the digital shadow of a production plant layout and its added value, initially a production scenario was virtually planned, physically implemented, and the real status of the layout was captured using the IPS. The scenario was initially virtually designed with the taraVRbuilder 2023 softwarefrom Tarakos. After the planning process was completed, the layout was set up in the learning factory of the ESB Business School on the campus of Reutlingen University. After setting up the production layout and during production, the physical layout was continuously captured and transferred to the digital space using the developed IPS. The added value generated by the automatic layout capture from the different stakeholder perspectives is described in the following sections.

### 5.3. Perspective of the Factory Planner

In this case, the factory planner was responsible for the basic layout planning. Subsequently, this person should keep the factory simulation up to date by adjusting the real positions of the plants in the simulation software. In addition, the task includes adding and updating further simulation values in consultation with the other stakeholders involved. This is to enable the factory simulation software to simulate different turbulences, such as fluctuating incoming orders. In this way, various “what-if” analyses can be carried out to determine the optimal configuration of the production processes and predict potential problems.

**Figure 17:**
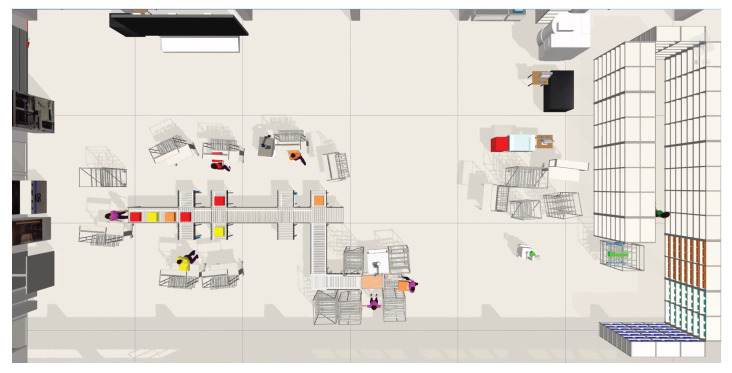
Updated plant layout in the simulation software.

In order to be able to perform a detailed simulation, it is necessary to record the real status of the plant layout and to update it in the simulation software. The actual state of the layout was recorded manually by the factory planner and transferred to the simulation software. The updated plant layout in the simulation software taraVRbuilder is shown in Figure 17.

A laser distance meter and a tape measure were used to measure the positions of the individual facilities. It turned out that the position measurement was relatively simple, but the determination of the orientation was relatively time-consuming. This was because it could not be measured directly and had to be calculated from distance measurements at several points on the facility. The subsequent manual transfer into the simulation tool was again quite quick and took only 5 min. However, the entire process took about 2.5 h.

In comparison, the recording of the layout with the IPS took about 6.5 min. A fully automatic update of the layout in the simulation software using the AutomationML data format was prepared but could not be tested because the import function had not yet been implemented. Therefore, the transfer of the measured poses also had to be done manually. Nevertheless, the whole process only took 12 min.

As can be seen in Table 1, the time saved for layout recording by using the IPS is very high compared to the manual approach.

### 5.4. Perspective of the Material Flow Analyst

In this scenario, the role of the material flow analyst includes the following tasks, such as recording the processing times of the products, which entails tracking the time a product has spent at a certain facility. In addition, the transport routes of the products throughout the individual production steps must be recorded. The recording process in material flow analysis is an extensive undertaking and therefore, depending on the complexity of the production system, the use of technical support systems is often necessary. Such systems are for example RFID tracking or barcode scanning, which are used at certain facilities to record individual process times. In principle, the layout has no influence on the classic determination of processing times. However, if more modern RTLS systems are used for material tracking in order to record the movements between the facilities in addition to the dwell times, the knowledge about the actual layout is essential. Otherwise it is not possible to allocate the measured material positions.

The actual added value of the digital shadow of the plant layout comes from the fact that in addition to the process and transport times, it is also possible to determine the transport speeds. This is achieved by knowing the real distances between the facilities through the measurement of the layout with the IPS in combination with the time records.

**Figure 18:**
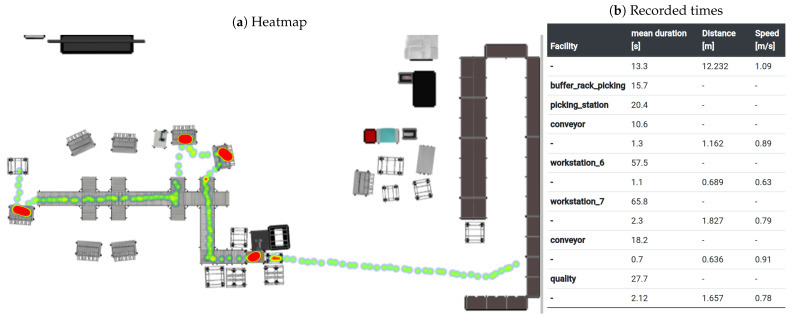
Sample output of the Telocate system with the updated layout. (**a**) Heatmap and (**b**) the recorded times as a table.

This means that the developed IPS has no advantage regarding effort in material flow analysis, but it enables the generation of additional information in combination with measured times. To demonstrate this, a software tool has been implemented to analyse the material positions measured by an ultrasonic based RTLS from the company Telocate in combination with the captured layout. Figure 18 shows a heat map that was created by tracking a product through the production process. In addition, on the right-hand side, the recording of the process and transport times as well as the calculated speeds are shown.

With the implemented use case, it could be shown how an updated plant layout can support the material flow analyst. Primarily, it serves as a visual support for the material flow analyst in tracing the material routes. By linking the output data of the developed IPS with the sensor data of an RTLS, it can be ensured that the underlying layout corresponds to reality, thus preventing the RTLS data from becoming useless in case of an unplanned layout change.

### 5.5. Perspective of the Route Planner

In this case, the route planner’s work involves programming a free navigating AMR. The purpose of this is to supply the individual workstations with the consumables needed for assembly. In order to enable such an AMR to travel certain routes and transport goods from a source to a target point, some preparations are necessary.

**Figure 19:**
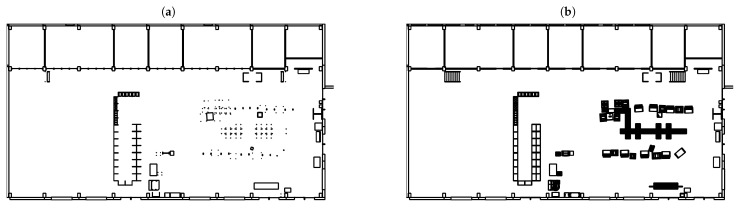
Generated (**a**) map and (**b**) costmap for the AMR.

After a change in the layout has occurred, the first thing to do is to record a new map of the environment. To do this, the AMR is steered through the entire factory using a joystick. During this movement, the AMR generates a map using the data provided by the built-in 2D laser scanner. The reason why this process has to be done manually is that the AMR can detect obstacles, but only those which are at the height of the laser scanner. As these are often only the legs of the facilities, a collision with for instance a conveyor belt can only be avoided by moving the AMR manually. During the recording process, some artefacts were caused in the map by people walking in the area; such obstacles that only occur briefly must be removed from the map. This first process took about 20 min in the implemented production scenario. Afterwards, so-called “no-go areas” have to be drawn in the created map. These are typically the boundaries of all facilities where a transit of the AMR would lead to a crash. The processing of the map took about 45 min in this case because the generated map almost only provides the legs of the facilities as reference points and therefore it had to be repeatedly remeasured. This means that after recording the environment with the built-in 2D laser scanners, a map is obtained that roughly corresponds to Figure 19a. Based on this map, the contours of the facilities have to be drawn manually to obtain a costmap according to Figure 19b. As the contours can be complex depending on the facility shape, the time required for the drawing is accordingly high. In the last step, the source and target poses have to be defined. To do this, the AMR was moved again with the joystick and driven to the desired points in the factory to save these poses. In the last step, the source and target poses must be defined. To do this, the AMR was again moved with the joystick and driven to the desired points in the factory to save these poses, which took about 10 min.

**Figure 20:**
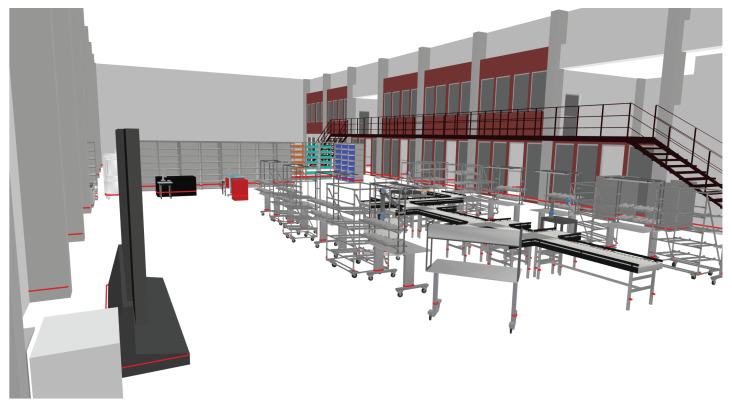
Generated 3D model of the plant layout using the IPS.

Instead of using this manual approach to prepare the AMR, the maps can also be created from the data generated by the IPS. The AMR requires the maps in the form of bitmap files. There is a map which serves as orientation for the AMR within the plant and a so-called costmap which contains the no-go areas. As the IPS creates a 3D model of the entire plant layout as shown in Figure 20, this model must be converted to the required maps.

To create the orientation map, a cut through the 3D model is made parallel to the ground at the height of the laser scanner on the real AMR, in this case the height is 21 cm. The result is a map in the form of a bitmap image, as shown in Figure 19a.

Here one can see that, for example, walls and shelves are shown as continuous lines and the legs of the tables and conveyor belts are shown as dots. Therefore, if only this map is used, it could lead the AMR to plan routes through a facility. To avoid this behaviour, a cost map is generated from the 3D model, which includes the obstacles from the floor to the height of the AMR. The cost map generated in this way is shown in Figure 19b, in which the contours of the individual facilities can be recognised. The AMR interprets the black parts of the map as no-go areas and would never plan a route through the facilities.

In contrast to the manual process, the map including the source and target poses was generated by the IPS by capturing the layout and transferred to the AMR fully automated within a few minutes. To enable this, the source and target poses have been determined and linked in advance relative to the individual facilities. This allows us to automatically calculate the AMR poses for each future layout change. As can be seen in Table 2, the manual approach requires over an hour of preparation time for the AMR and only a few minutes with the IPS.

It could be shown that there is a high potential for the use of the digital shadow of the plant layout in the route planning for free navigating AMR. The entire manual setup effort could be automated. This results in a significantly earlier start of production since there is no longer a need to wait for the AMR to be set up. In addition, the AMR constantly adapts to the changing conditions by continuously capturing the layout through the IPS.

## 6. Summary and Conclusions

The presented camera-based indoor positioning system was tested for the accuracy of the facility pose measurement as well as the recording duration using the test series in Section 4.4. In summary, an accuracy of less than 47 mm could be achieved in the position measurement. The maximum error in the angle measurement was below a value of ±1°. The required recording time was determined in relation to the number of facilities included and is less than 10 min for 50 facilities, for example.

In addition, the application of the entire system was tested on the basis of a use case. It has been demonstrated that significant time savings for stakeholders can be achieved in the recording of the plant layout. This is not only due to the measurement procedure itself, but also to the data conversion into the corresponding formats for the exemplary software tools. A particular advantage of the generated digital shadow of the plant layout is the automatic map generation for AMRs. As these are directly involved in the production process and their set-up phase can delay the production, the greatest potential is found here in the application of the proposed indoor positioning system.

The proposed system enables the creation of a digital shadow of plant layouts of sufficient quality for the intended use cases. As only a PTZ camera and AprilTags printed on paper are used for the data capturing, this system can be realised very cost-effectively. Compared to other positioning systems, which are often much more expensive and require a considerable higher effort for maintenance and installation, good results could be achieved with this system. While the development of the IPS and the creation of a digital shadow of the plant layout have provided valuable insight and practical implications, it is important to acknowledge certain limitations that could be addressed in future research. In addition, there are opportunities for further work to improve upon the current results. The implementation of the scanning strategy with the PTZ camera was relatively simple by running a fixed sequence of angle settings. However, there is potential for further improvement by incorporating smarter algorithms or using machine learning approaches. By collecting historical data, it may be possible to develop an algorithm that can adaptively scan areas where the layout changes more frequently. This intelligent approach would scan these more dynamic areas with a higher frequency and potentially optimise the total scan times. In this study, the main focus was on the arrangement of facilities, but for future research, the distribution of human resources should also be investigated. Another suggestion for future work could be the collection of all historical data about the plant layout, material flow, and routes in a unified format to automatically create new layout proposals using artificial intelligence.

## Figures and Tables

**Table 1:** Duration of the layout capture processes.

Layout Capture Method	Layout Recording Process	Transfer to Simulation Software	Total Time
Manual approach	2.5 h	5 min	≈2.5 h
IPS	6.5 min	5 min	≈12 min

**Table 2:** Duration of the AMR setup processes.

AMR Setup Method	Map Generation	Drawing the No-Go Areas	Creating Source and Target Poses	Total Time
Manual approach	20 min	45 min	10 min	≈1.25 h
IPS	6.5 min	0 min	0 min	≈6.5 min

## Data Availability

Data sharing not applicable.
